# Outcomes reported in trials of treatments for severe malaria: The need for a core outcome set

**DOI:** 10.1111/tmi.13803

**Published:** 2022-08-21

**Authors:** Lamprini Lampro, Elizabeth C. George

**Affiliations:** ^1^ Medical Research Council Clinical Trials Unit at University College London London UK; ^2^ Intensive Care National Audit and Research Centre London UK

**Keywords:** core outcome set, outcome measures, severe malaria, systematic review, treatment

## Abstract

**Objectives:**

Malaria is one of the most important parasitic infectious diseases worldwide. Despite the scale‐up of effective antimalarials, mortality rates from severe malaria (SM) remain significantly high; thus, numerous trials are investigating both antimalarials and adjunctive therapy. This review aimed to summarise all the outcome measures used in trials in the last 10 years to see the need for a core outcome set.

**Methods:**

A systematic review was undertaken to summarise outcomes of individually randomised trials assessing treatments for SM in adults and children. We searched key databases and trial registries between 1 January 2010 and 30 July 2020. Non‐randomised trials were excluded to allow comparison of similar trials. Trial characteristics including phase, region, population, interventions, were summarised. All primary and secondary outcomes were extracted and categorised using a taxonomy table.

**Results:**

Twenty‐seven of 282 screened trials met our inclusion criteria, including 10,342 patients from 19 countries: 19 (70%) trials from Africa and 8 (30%) from Asia. A large amount of heterogeneity was observed in the selection of outcomes and instruments, with 101 different outcomes measures recorded, 78/101 reported only in a single trial. Parasitological outcomes (17 studies), neurological status (14 studies), death (14 studies) and temperature (10 studies), were the most reported outcomes. Where an outcome was reported in >1 study it was often measured differently: temperature (4 different measures), renal function (7 measures), nervous system (13 measures) and parasitology (10 measures).

**Conclusion:**

Outcomes used in SM trials are inconsistent and heterogeneous. Absence of consensus for outcome measures used impedes research synthesis and comparability of different interventions. This systematic review demonstrates the need to develop a standardised collection of core outcomes for clinical trials of treatments for SM and next steps to include the development of a panel of experts in the field, a Delphi process, and a consensus meeting.

## INTRODUCTION

Malaria is one of the most important parasitic infectious diseases worldwide. The vast majority of all malaria cases (>90%) are attributable to *Plasmodium falciparum*, which produces high levels of parasitaemia. This subsequently causes sequestration of mature‐staged infected erythrocytes in the microvasculature of organs, resulting in micro‐circulatory ischaemia and multi‐organ failure [1]. Severe or complicated malaria is the most life‐threatening and potentially fatal manifestation of the disease and most cases will develop severe neurological deficit or death in the absence of prompt and effective management [1, 2].

Despite the efforts and progress over the past years in reducing malaria incidence and mortality, since 2015 the disease control has levelled off [3, 4]. The estimated global incidence of severe malaria (SM) reaches 2 million reports every year [2], with children under 5 years and pregnant women living in sub‐Saharan Africa being the most vulnerable groups [3, 5]. Even though the standard treatment of artesunate following the landmark SEAQUAMAT [6] and AQUAMAT [7] trials has improved outcomes in SM, the mortality rates remain significantly high, with rates ranging from 10% to 20% for in‐hospital care and up to 100% for patients unable to reach health facilities [8, 9]. The heaviest burden falls on children from resource limited countries where very few will ever reach a high dependency or intensive care unit to receive specialised treatment [10]. The global emerging need for effective treatment strategies alongside antimalarials has also led to research in adjunctive treatments to improve clinical outcomes and a number of randomised controlled trials (RCTs) in adjunctive therapy in order to treat SM successfully [11].

A critical component in measuring and evaluating antimalarial or adjunctive treatment efficacy is the selection of appropriate outcomes for the RCTs [11, 12]. However, to date there is no agreed uniform method to assess the efficacy of antimalarials or adjunctive treatments and no conclusive analysis of outcomes being used in this area has been published. In addition to that, there is a growing recognition that the lack of consensus with regard to the outcomes measured in trials leads to inconsistencies and high heterogeneity between reported outcomes [13]. Furthermore, only comparable and standardised outcomes can be pooled in meta‐analyses, providing meaningful trial comparisons and informing clinical practice.

A standardised minimum set of outcomes, known as a Core Outcome Set (COS), would help define clinically meaningful outcomes for the treatment of SM, facilitate transparency and reduce outcome reporting bias, while enhancing the credibility and validity of future trials [13, 14, 15, 16]. Researchers are not restricted to the outcomes recommended in the COS, but rather use as a primary outcome those outlined in the COS, and continue exploring other relevant and important health outcomes, which may be at different points along the clinical pathway to serious illness and mortality [13, 14].

The aim of this study is to describe the outcomes reported in individually randomised trials investigating treatments for SM in adults and children. This systematic review of trials in this area over the last 10 years will help further demonstrate the need for a core set and also provide a comprehensive synthesis of all outcomes published.

## METHODS

### Search strategy

A systematic review in line with the 2009 Preferred Reporting Items for Systematic Reviews and Meta‐Analyses statement (PRISMA) guidelines was performed [17]. Planned, ongoing or completed interventional RCTs evaluating treatments for SM were considered eligible. Three key databases and three clinical trial registries were searched (May 2020), including ‘Cochrane Central Register of Controlled Trials’ (CENTRAL) published in The Cochrane Library, MEDLINE (OVID), ‘Literatura Latino Americana em Ciências da Saúde’ (LILACS), ‘International Standard Randomised Controlled Trial Number’ (ISRCTN), ClinicalTrials.gov and ‘Pan‐African Clinical Trial Registry’ (PACTR). In addition, the citation lists of retrieved trials were checked for additional eligible records.

Inclusion criteria were RCTs between 2010 and 2020 involving only hospitalised adults or children with SM (including cerebral malaria) following the WHO definition [2], and comparing antimalarial or adjunctive treatments for SM. Observational studies, literature reviews, secondary analyses of RCTs, non‐randomised trials, prevention or vaccine studies, trials including both severe and uncomplicated malaria, non‐human trials, conference abstracts, and posters were excluded.

The search terms were developed on the basis of four key concepts: SM, treatment, hospital and Randomised Controlled Trial. The search was limited to trials published in the English language. The time period restriction was to provide a comprehensive snapshot view of the outcomes from trials published in the last decade. A full list of search terms, concept construction and data extraction processes are provided in Table S1.

### Data extraction and analysis

**FIGURE 1:**
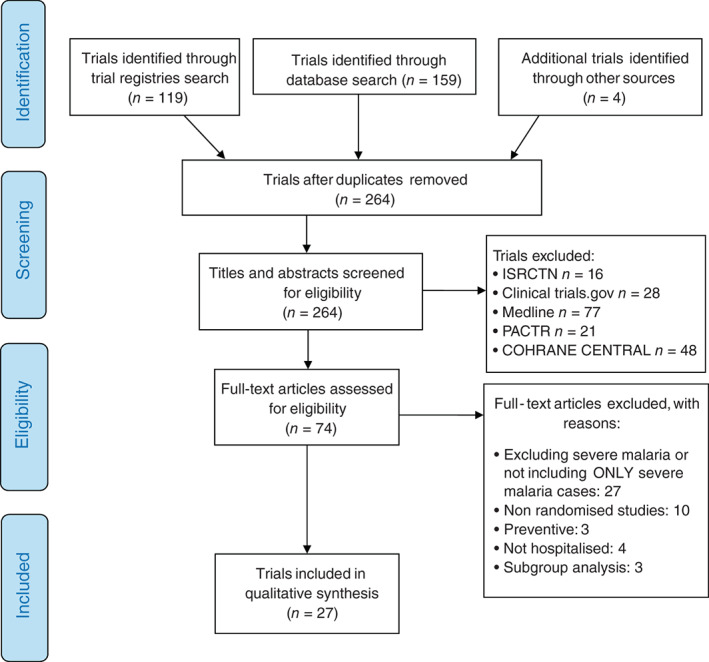
PRISMA flowchart

The primary reviewer (LL) double‐screened potentially relevant records based on titles and abstracts, and reviewed the full text of selected trials to assess for eligibility. Data from eligible RCTs was then extracted and entered into tables created in Microsoft Word (Table S2). Eligible trials identified through the trial registries that did not include published results, were cross‐checked using registration number, primary investigator name, and country of study against the published studies in order to screen‐out duplicates. Trials requiring further review were discussed and resolved by consensus with the second reviewer (ECG). Trials that did not meet the inclusion criteria were excluded with reasons outlined in the PRISMA flowchart (Figure 1).

All primary and secondary outcome measures from eligible trials were summarised and tabulated (Table S2). Descriptions of all outcome measures were extracted verbatim from the study protocols or trial registries. Primary outcome measures that were not defined clearly in the trial publication were identified from the sample size calculations. The instruments used for the quantification of each outcome were also captured. The outcome measures were then grouped and categorised into the domains of the outcome taxonomy [15] recommended by the ‘Core Outcome Measures in Effectiveness Trials’ (COMET) initiative [14].

## RESULTS

Our search retrieved 159 records from electronic bibliographic database searches and 119 from trial registries (Figure 1). Four additional trials were identified from the citation lists of the retrieved papers. After removing 18 duplicate trials, 264 unique records were processed through title and abstract screening. One hundred and ninety studies were then excluded after screening title and abstract (if published) or study characteristics (on trial registries). Finally, 74 full‐text studies were comprehensively reviewed, resulting in 27 records that met the inclusion criteria for this review (Figure 1).

### Included studies

A total of 10,342 patients with SM were included in 27 trials [7, 18, 19, 20, 21, 22, 23, 24, 25, 26, 27, 28, 29, 30, 31, 32, 33, 34, 35, 36, 37, 38, 39, 40, 41, 42, 43], of which 7702 patients were included in trials testing primary antimalarial therapies and 2640 in trials testing adjunctive therapies in addition to standard antimalarial treatment. The majority of the trials conducted in the last decade studied the role of adjunctive therapy in the management of SM (*n* = 18, 67%). The trial interventions are classified as drug‐related in 26 studies [7, 18, 20, 21, 22, 23, 24, 25, 26, 27, 28, 29, 30, 31, 32, 33, 34, 35, 36, 37, 38, 39, 40, 41, 42, 43] and device‐related in only one study [19]. The control group was a placebo or no intervention in 12 trials (44%) and active treatment in 13 trials (56%). Two further trials [23, 25] had dose‐comparison concurrent control. Fifteen (56%) trials included patients aged 3 months‐15 years and 12 (44%) included patients of all ages. Overall, 18 trials were open‐label and 7 used blinding; 2 further trials did not report any blinding methods. Full details with study characteristics are provided in Table S2.

**FIGURE 2:**
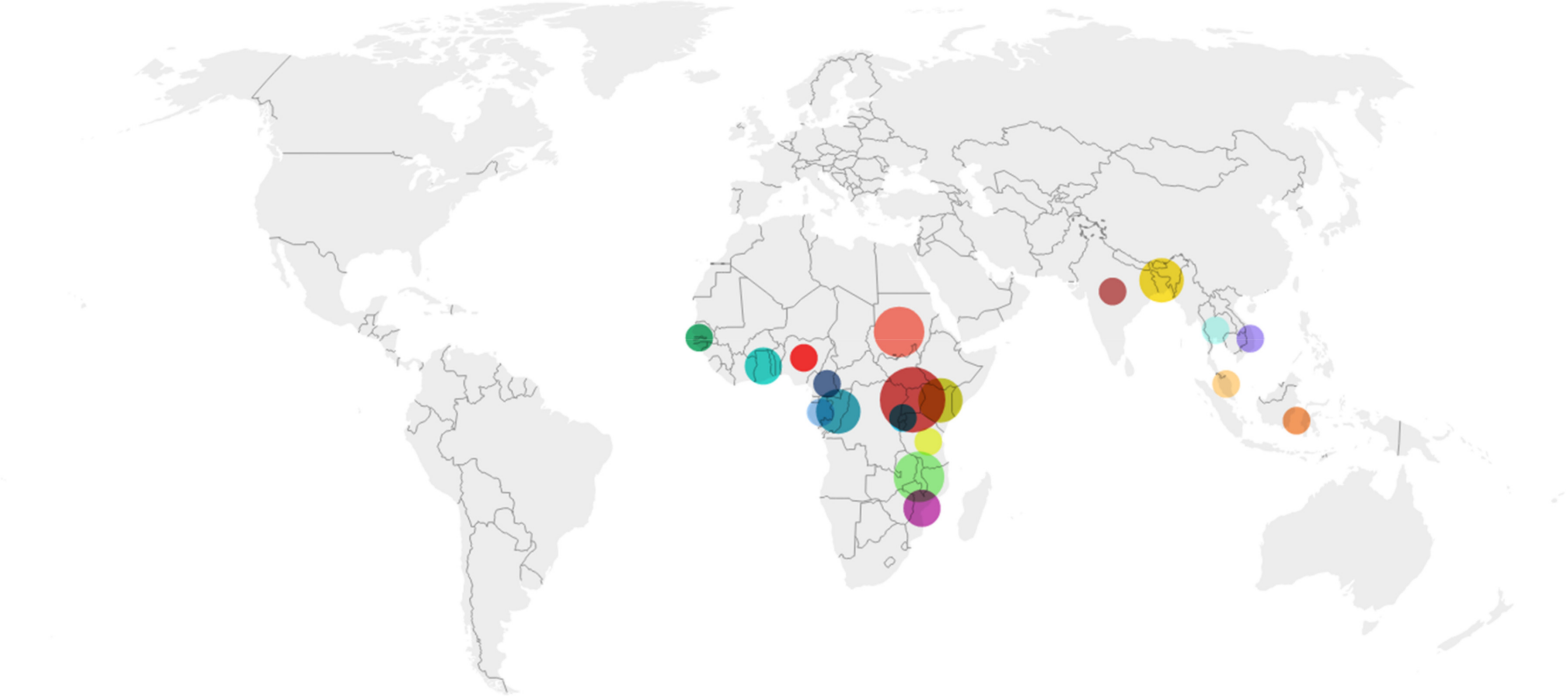
Map of severe malaria trial sites across the world. 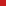 Uganda (*n* = 7), 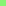 Malawi (*n* = 4), 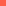 Sudan (*n* = 3), 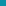 Congo (*n* = 3), 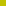 Kenya (*n* = 3), 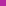 Mozambique (*n* = 2), 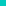 Ghana (*n* = 2), 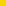 Bangladesh (*n* = 3), 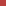 India (*n* = 1), 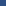 Cameroon (*n* = 1), 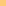 Malaysia (*n* = 1), 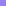 Vietnam (*n* = 1), 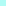 Thailand (*n* = 1), 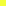 Tanzania (*n* = 1), 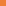 Indonesia (*n* = 1), 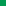 Gambia (*n* = 1), 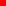 Nigeria (*n* = 1), 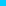 Rwanda (*n* = 1), 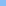 Gabon (*n* = 1). The Democratic Republic of the Congo (DRC) and the Republic of Congo are grouped together under Congo

As shown in Table 1 and Figure 2, the highest proportion of studies was conducted in sub‐Saharan Africa (*n* = 19, 70%). Three additional trials were multi‐centre enrolling patients across several sub‐Saharan countries. Most of these studies (*n* = 11, 60%) were RCTs studying adjunctive therapies for the treatment of SM, while eight (*n* = 8, 40%) were on antimalarial treatments. Eight trials in total were conducted in Asia (*n* = 8, 30%), with 7 (88%) studying adjunctive therapies for SM and only 1 trial was on antimalarial treatment.

**TABLE 1:** Baseline characteristics of randomised controlled trials in treatment of severe malaria

	Antimalarial therapy	Adjunctive therapy	Total
Year	*n* = 9 (33%)	*n* = 18 (67%)	*n* = 27
Trial participants	7702	2640	10,342
2010–2015	7	12	19 (70%)
2016–2020	2	6	8 (30%)
Phase			
Phase I/II	‐	2	2 (7%)
Phase II	5	12	17 (63%)
Phase III	4	4	8 (30%)
Phase IV	‐	‐	
Region			
Sub‐Saharan Africa	8	11	19 (70%)
Asia	1	7	8 (30%)
Comparator intervention			
Active	8	5	13 (48%)
Placebo or no intervention	0	12	12 (44%)
Dose‐comparison control	1	1	2 (7%)
Age range			
3 months–15 years	4	11	15 (56%)
Patients of all ages	5	7	12 (44%)

As summarised in Table 1, nine RCTs in the period 2010–2020 were studying antimalarial drug treatments for SM [7, 18, 25, 27, 29, 31, 37, 38, 40]. The largest proportion (7/9) of the antimalarial trials studied the efficacy of intravenous artesunate over intravenous quinine for the treatment of SM, one phase 2 trial checked the bioavailability of rectal artesunate and one other checked the superiority of intramuscular artesunate over intramuscular artemether.

The majority (*n* = 18, 67%) of SM trials conducted in the period 2010–2020 studied the role of adjunctive treatments for the management of SM (Table 1) [19, 20, 21, 22, 23, 24, 26, 28, 30, 32, 33, 34, 35, 36, 39, 41, 42, 43]. The adjunctive treatments that were tested in these trials were: azithromycin, paracetamol (2 trials), paracetamol versus ibuprofen, hypertonic saline versus mechanical ventilation, rosiglitazone, levetiracetam, iron supplements, nitric oxide (2 trials), dextran versus hydroxyethyl starch, enteral feeding, levamisole, vitamin A, mannitol, ursoxycholic acid and l‐arginine. However, to date none of the published studies have shown clear efficacy results while a few are still ongoing.

### Outcomes assessed

**FIGURE 3:**
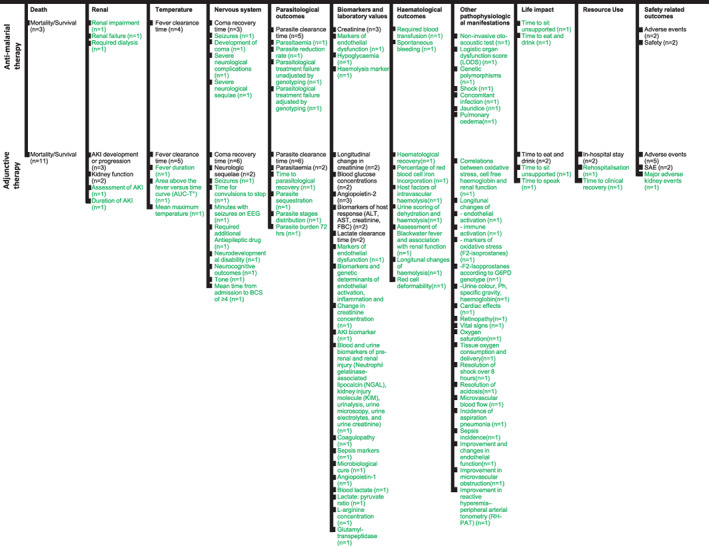
Hierarchical representation of outcomes recorded in 27 severe malaria trials. Outcomes ordered by domain. Numbers in brackets illustrates number of studies reporting outcome. All outcomes corresponding to each study are listed in full in appendix. Outcomes in green reported in only a single study. AKI, acute kidney injury; BCS, Blantyre Coma scale; EEG, electroencephalogram; FBC, full blood count; SAE, serious adverse events. Figure design taken from Webbe et al. [44]

Overall, 101 distinct outcomes are evaluated in the included trials, and were inconsistently reported and measured among the 27 RCTs (Figure 3). Twenty‐three (85%) of the trials pre‐defined their primary and secondary outcomes. We identified 11 generic outcome categories, ordered following the taxonomy [15]. Death was a frequent outcome domain measured in 14/27 trials, with 11 of them testing adjunctive therapies. Parasite clearance time, neurological abnormalities and fever, the most commonly assessed outcomes, were only assessed in 17, 14 and 10 studies, respectively. All remaining outcomes were evaluated in less than half of the trials, and 78/101 were only reported in a single trial, highlighting an important heterogeneity in outcomes selection. The least reported outcomes were life impact and resource use.

Studies reported using a variety of methods, sometimes in combination, to measure the outcomes within the same outcome domain. Also, each of the outcome domains included different and inconsistent outcome measures across the trials and more than 90 outcomes appeared only once or twice (Figure 3). The highest inconsistency was seen in the biomarkers/laboratory values domain, where 20 unique outcome measures were used.

Where trials had reported the same outcome, for example seizures, these may not be comparable as different outcome measures were used; for example, time for convulsions to stop after initiating treatment [34], minutes with seizures on electroencephalogram (EEG) 72 h after treatment allocation [39], or seizures detected clinically or on daily EEG during the study duration [24]. Renal function was measured using 7 outcomes: Acute Kidney Injury (AKI) development, AKI progression, AKI duration, Major Adverse Kidney Events (MAKE), Assessment of Acute Kidney Injury, haemodialysis trends, urine colour chart, creatinine concentration, and AKI biomarkers. Where trials reported the AKI definition, this was also different as one defined AKI as ‘creatinine ≥26.5 μmol/L or ≥1.5× baseline’ [43], while another defined it as ‘(i) an absolute increase in serum creatinine of >26.5 μmol/L from enrolment creatinine; (ii) a percentage increase in serum creatinine of >50% from enrolment; (iii) post‐enrolment onset of oliguria of less than 0.5 ml/kg/h for more than 6 h; (iv) 24 h urine output of <400 ml after rehydration and urinary obstruction excluded’ [30]. AKI development also differed in measurement timing: first 7 days of enrolment, over 72 h, and 14 days were used.

Outcome measures in fever comprised fever clearance time, mean maximum temperature, area above the fever versus time curve, and fever duration. Where fever clearance time was measured, the time frame included: during the first 7 days from enrolment, 72 h, and the time at which the axillary temperature first dropped below 37.5°C and remained below 37.5°C for 24 h. Parasite clearance time was defined and assessed also at different time points, including the first 7 days of enrolment, 72 h, time (in h) from the onset of treatment to the time of the first of two successive negative blood smears through hospital discharge, first 24 h after initiating treatment, start of the treatment until the first negative blood film, with the blood film then remaining negative for 24 h (Figure 3, Table S3).

Furthermore, same phase trials measured mortality distinctively; for instance mortality within 48 h post hospital admission and 14 days post hospital admission [36], mortality within 7 days of randomisation [19], mortality within 4 weeks [42], mortality at 48 h [20], mortality at 48 days and 90 days [23].

Among 27 included studies and after reviewing published papers and protocols where available, we found that no study reported patient or public involvement in outcome selection.

## DISCUSSION

This study is, to our knowledge, the first comprehensive systematic review of outcome measures used in RCTs studying treatments for SM, summarising data from 27 trials [7, 18, 19, 20, 21, 22, 23, 24, 25, 26, 27, 28, 29, 30, 31, 32, 33, 34, 35, 36, 37, 38, 39, 40, 41, 42, 43] and 10,342 patients. Our review shows that the focus over the last 10 years for the management of SM is on adjunctive drug treatments. To date, parenteral artesunate remains the standard antimalarial treatment for SM, while no new antimalarial agents have been investigated in an RCT. Importantly, we found that although SM still accounts for substantial morbidity and mortality worldwide and there are trials in the pipeline for SM treatments, we highlight that there is no uniform method of measuring outcomes in this area, which subsequently results in many different and heterogeneous outcomes.

The highest proportion of SM trials between 2010 and 2020 were conducted in sub‐Saharan Africa (*n* = 19, 70%), where most of the cases are reported. Although children under 5 years of age are the most vulnerable group affected by SM [5, 10, 45], our findings suggest that the past decade's trials were not focused entirely on the pediatric population, with 15/27 (56%) of the SM trials enrolling patients aged 3 months‐15 years. One explanation could be that few of the children with SM will reach health centres and be cared for in specialised units, so most will die before reaching hospital facilities [46, 47, 48]. Also, the sociocultural differences, values and beliefs around medical research, and barriers of obtaining informed consent in critically ill paediatric population, are only some of the factors hindering clinical trials in socioeconomic deprived regions such as sub‐Saharan Africa and Asia [49, 50]. Lastly, despite the substantial morbidity and mortality of maternal SM [51], pregnant women were not included in randomised trials of treatment drugs or adjunctive therapy over the past decade, and therefore there would need to be careful consideration of the generalisability of findings onto this population [52].

This review aimed to identify all outcomes that have been used in RCTs studying treatments for SM in order to inform the design and development of a COS in light of the COMET initiative [14]. For this reason, we summarised all the primary and secondary outcomes of published and unpublished RCTs studying the treatment of SM. While the selected trials for analysis are comparable to each other with regards to their design, scope and patient inclusion criteria, as they are all individually randomised RCTs studying treatment options for patients with SM in hospital settings, the outcome measures showed wide variability. Due to this, we attempted to organise and group the outcome measures into main outcome domains by using the outcome taxonomy [14] recommended by the COMET initiative [15].

This review quantifies the high degree of inconsistency and heterogeneity around outcome measures used SM trials (Figure 3). This could be explained by the fact that malaria is a disease that affects multiple organs and hence different treatments are targeting different outcome domains. However, it should be noted that the large variability was not only evident between the outcome domains across trials, but within each subdomain too. More precisely, outcomes such as parasite clearance time, neurological abnormalities and fever were inconsistently measured and reported at multiple time points (which were often poorly specified). In addition, even an outcome as crucial as survival has not been universally reported. Heterogeneity of outcome selection is further illustrated by the large number of outcome measures only reported in a single trial (78/101), which reflects the high degree of variety between outcomes measured and reported in SM.

Therefore, the heterogeneity between the selected outcomes, as well as how the outcomes were measured and reported, identifies a clear obstacle to evidence synthesis. This finding is not something new in clinical research as several reports have previously highlighted this as a major problem [13, 16, 53]. Various areas of health have had a COS developed and successfully implemented, particularly in rheumatology and chronic pain [54, 55], however, to date no work has been done in SM. Using a validated and standardised tool such as COS in this area, would limit outcome reporting biases, prevent research waste, fewer trials would have to be excluded in meta‐analyses and less heterogeneity would be seen in reported outcomes [13, 14]. Also, the COS includes the ‘minimum set of outcomes’, and therefore researchers are still able to explore other relevant and important health outcomes [13]. The outcome measures summarised in this review can be used as the starting point and catalyst for the development of a COS.

Like sepsis, SM has a complex clinical presentation including several complications [10], that require a range of targeted adjunctive treatments which poses a challenge to standardising outcomes. This challenge has been discussed with experts from the Severe Malaria—A Research and Trials (SMAART) Consortium (funded by the Wellcome Trust [209265/Z/17/Z]) and they noted that part of the development of new therapies targeting each complication requires Phase II trials. These smaller trials require endpoints that lead to different outcomes that reflect the treatment's impact on the organ or syndrome that is being targeted, as clinical surrogate outcomes for prognosis. However, it has been noted that even if surrogate endpoints are being used to help address the issue of underpowered studies, mortality should always be measured as a secondary endpoint [56]. These trials also are not often able to capture continuing mortality and morbidity during the months following admission for SM. For antimalarials, lactate concentration was found to be a valid surrogate endpoint at 8 or 12 h after admission if studies were aiming to improve microcirculation, but measures of coma recovery were not valid as surrogates for mortality [57]. The validation of biomarkers as surrogate endpoints is challenging, as meta‐analyses of many trials of drugs in the same class are required measuring both the surrogate and the clinical endpoint [57], which may also have delayed the assessment of the need for a COS. To develop a COS evidence for the validity of surrogate outcomes would also need to be reviewed in any consensus meeting along with definitions and how each would be measured using the guidance on developing outcome measurement instruments [58]. Any Delphi process to create a COS would need to be focussed on outcomes and how to standardise measurement and definitions across the different clinical syndromes, rather than research processes or impact as has been done previously [59].

A key strength of our review was the comprehensive synthesis of evidence around outcomes used in SM trials, which goes beyond anything previously published in the literature of this field. This analysis provides also a descriptive assessment of the current portfolio of all RCTs that have been done in SM over the last decade, including phases, population characteristics, region, treatment features, and outcome measures. This review adhered to systematic review methods, including screening a wide range of electronic bibliographic databases as well as clinical trial registries, and appraising a large body of published and unpublished trials from 13 countries. By comprehensively searching the trial registries we were able to assess all the current registered trials, despite their publication status and therefore we aimed to minimise publication bias. Lastly, the inclusion and exclusion criteria were built around the WHO definition for SM, which utilised a standardised format in order to summarise data from similar studies and allow fair comparisons.

Our review has limitations. We did not examine other types of literature such as reviews nor certain types of studies, such as observational studies and non‐randomised trials. However, their exclusion allowed extrapolation of conclusions from comparing similar trials. Furthermore, the restriction to English language trials does have the risk of missing some trials. The restriction of studies from the 10‐year period 2010 to 2020 is also a potential limitation and some information on outcomes from trials published prior to this may have been missed. Finally, the included trials were assessed for relevance by only a single person, however, uncertainties and missing information were resolved by consensus with the author ECG.

## CONCLUSION

To the best of our knowledge, this is the first study to systematically review all outcomes measured in RCTs of SM treatment and inform the need for a COS in this field. Given the lack of universal adoption of a minimum set of core outcomes and the consequently high variability in outcomes measured, it becomes evident that development of a COS for SM treatment is an urgent task. Also, since SM has a large incidence globally, these results are crucial and need to be highlighted in order to conceive future work; especially given the trials in the pipeline of drug development which would benefit from a COS. Next steps include the development of a panel of experts in the field, a Delphi process, and a consensus meeting. Lastly, a key stage in the development of a high quality COS would include established international collaboration between key stakeholders, including health professionals, researchers, and patient representatives, in the selection and specification of these outcomes [13, 14, 60].

## FUNDING INFORMATION

Funding for ECG is provided by the Medical Research Council as core support to the MRC CTU at UCL (MC_UU_00004/05).

## Supporting information


**Appendix S1** Supporting Information
