# Supplementary material for: Outcomes reported in trials of treatments for severe malaria: The need for a core outcome set
Source: Trop Med Int Health. 2022 Aug 21;27(9):767–75. doi: 10.1111/tmi.13803 (PMC9545330; doi:10.1111/tmi.13803)
Supplement: Supplementary file 1 — Appendix S1 Supporting Information [file TMI-27-767-s001.docx]

**Appendix**

| **Supplementary Table 1: Search strategy** |
| --- |
| **CENTRAL search strategy**  #1 MeSH descriptor: [Malaria] explode all trees and with qualifier(s): [drug therapy - DT, therapy - TH] 1442  #2 MeSH descriptor: [Malaria, Cerebral] explode all trees and with qualifier(s): [drug therapy - DT, therapy - TH] 63  #3 MeSH descriptor: [Malaria, Falciparum] explode all trees and with qualifier(s): [drug therapy - DT, therapy - TH] 923  #4 MeSH descriptor: [Malaria, Vivax] explode all trees and with qualifier(s): [drug therapy - DT, therapy - TH] 116  #5 ("cerebral malaria"):ti,ab,kw with Publication Year from 2010 to 2020, in Trials with 'Infectious Diseases' in Cochrane Groups (Word variations have been searched) 13  #6 ("SEVERE malaria"):ti,ab,kw with Publication Year from 2010 to 2020, in Trials with 'Infectious Diseases' in Cochrane Groups (Word variations have been searched) 47  #7 #1 OR #2 OR #3 OR #4 OR #5 OR #6 with Publication Year from 2010 to 2020, in Trials 491  #8 MeSH descriptor: [Therapeutics] explode all trees 303480  #9 #7 AND #8 with Publication Year from 2010 to 2020, in Trials 210  #10 (prevent*):ti,ab,kw with Publication Year from 2010 to 2020, in Trials with 'Infectious Diseases' in Cochrane Groups (Word variations have been searched) 639  #11 (net*):ti,ab,kw with Publication Year from 2010 to 2020, in Trials with 'Infectious Diseases' in Cochrane Groups (Word variations have been searched) 120  #12 (prophyla*):ti,ab,kw with Publication Year from 2010 to 2020, in Trials with 'Infectious Diseases' in Cochrane Groups (Word variations have been searched) 99  #13 (vaccine*):ti,ab,kw with Publication Year from 2010 to 2020, in Trials with 'Infectious Diseases' in Cochrane Groups (Word variations have been searched) 248  #14 ("diagnostic test*"):ti,ab,kw with Publication Year from 2010 to 2020, in Trials with 'Infectious Diseases' in Cochrane Groups (Word variations have been searched) 111  #15 ("uncomplicated"):ti,ab,kw with Publication Year from 2010 to 2020, in Trials with 'Infectious Diseases' in Cochrane Groups (Word variations have been searched) 221  #16 #10 OR #11 OR #12 OR #13 OR #14 OR #15 with Publication Year from 2010 to 2020, in Trials 1090  #17 #9 NOT #16 with Publication Year from 2010 to 2020, in Trials 129  #18 (hospital*):ti,ab,kw with Publication Year from 2010 to 2020, in Trials with 'Infectious Diseases' in Cochrane Groups (Word variations have been searched) 260  #19 (inpatient*):ti,ab,kw with Publication Year from 2010 to 2020, in Trials with 'Infectious Diseases' in Cochrane Groups (Word variations have been searched) 12  #20 (patient*):ti,ab,kw with Publication Year from 2010 to 2020, in Trials with 'Infectious Diseases' in Cochrane Groups (Word variations have been searched) 801  #21 #18 OR #19 OR #20 901  #22 #17 AND #21 with Publication Year from 2010 to 2020, in Trials 18  #23 #9 AND #21 64 |
| **MEDLINE (Ovid) search strategy**   \| **#** \| **Searches** \| **Results** \| \| --- \| --- \| --- \| \| 1 \| Malaria/dt, th [Drug Therapy, Therapy] \| 10122 \| \| 2 \| Malaria, Cerebral/dt, th [Drug Therapy, Therapy] \| 486 \| \| 3 \| Malaria, Falciparum/dt, th [Drug Therapy, Therapy] \| 6009 \| \| 4 \| Malaria, Vivax/dt, th [Drug Therapy, Therapy] \| 1107 \| \| 5 \| (severe adj3 malaria).tw. \| 4604 \| \| 6 \| 1 or 2 or 3 or 4 or 5 \| 20170 \| \| 7 \| treatment*.mp. [mp=title, abstract, original title, name of substance word, subject heading word, floating sub-heading word, keyword heading word, organism supplementary concept word, protocol supplementary concept word, rare disease supplementary concept word, unique identifier, synonyms] \| 5170233 \| \| 8 \| therap*.mp. [mp=title, abstract, original title, name of substance word, subject heading word, floating sub-heading word, keyword heading word, organism supplementary concept word, protocol supplementary concept word, rare disease supplementary concept word, unique identifier, synonyms] \| 6216150 \| \| 9 \| "drug therap*".mp. [mp=title, abstract, original title, name of substance word, subject heading word, floating sub-heading word, keyword heading word, organism supplementary concept word, protocol supplementary concept word, rare disease supplementary concept word, unique identifier, synonyms] \| 2321385 \| \| 10 \| "drug treatment*".mp. [mp=title, abstract, original title, name of substance word, subject heading word, floating sub-heading word, keyword heading word, organism supplementary concept word, protocol supplementary concept word, rare disease supplementary concept word, unique identifier, synonyms] \| 37595 \| \| 11 \| 7 or 8 or 9 or 10 \| 8613607 \| \| 12 \| 6 and 11 \| 17999 \| \| 13 \| "randomized controlled trial".pt. \| 514078 \| \| 14 \| (random$ or placebo$ or single blind$ or double blind$ or triple blind$).ti,ab. \| 1273723 \| \| 15 \| (retraction of publication or retracted publication).pt. \| 16711 \| \| 16 \| 13 or 14 or 15 \| 1389587 \| \| 17 \| (animals not humans).sh. \| 4705549 \| \| 18 \| ((comment or editorial or meta-analysis or practice-guideline or review or letter) not "randomized controlled trial").pt. \| 4648328 \| \| 19 \| (random sampl$ or random digit$ or random effect$ or random survey or random regression).ti,ab. not "randomized controlled trial".pt. \| 93562 \| \| 20 \| 16 not (17 or 18 or 19) \| 1019653 \| \| 21 \| 12 and 20 \| 1663 \| \| 22 \| limit 21 to (english language and yr="2010 -Current") \| 695 \| \| 23 \| (Hospital* or inpatient*).tw. \| 1363580 \| \| 24 \| limit 23 to (english language and yr="2010 -Current") \| 644222 \| \| 25 \| 21 and 23 and 24 \| 95 \| \|  \| The search filter for RCTs was found online, at the British Medical Journal (BMJ) best practice website.^75^ \|  \| |
| **LILACS Database search strategy**  Search term: malaria (title, abstract, subject)  Type of study: Controlled clinical trial  Language: English  Publication year range: 2010-2020 |
| **ISRCTN search strategy**  “severe malaria” OR (“cerebral malarial”) within condition: Malaria  Interventions: Treatment OR therapy OR drug OR medication  Trial start date: from: 01/01/2010 to 30/07/2020  Condition category: Infections and infestations |
| **ClinicalTrials.gov search strategy**  (Severe OR cerebral OR falciparum OR vivax)  AND  (hospital OR patient OR inpatient)  And  (treatment OR therapy OR drug OR medication)  NOT  (uncomplicated)  NOT  (prevention OR vaccine OR diagnostic test OR net)  Category: Interventional studies  Start date 01/01/2010 to 30/07/2020 |
| **PACTR search strategy**  Search terms: malaria  Intervention: Supportive care OR Treatment: devices OR Treatment: Drugs OR Treatment: other OR Treatment: surgery  Allocation: Randomised  Registration date: 01/01/2010 to 30/07/2020  Trial Start date: 01/01/2010 to 30/07/2020 |

| **Supplementary Table 2: Detailed study characteristics of included trials** | | | | | |
| --- | --- | --- | --- | --- | --- |
| **Trial and publication/registration year** | **Participants** | **Design and Phase** | **Intervention** | **Primary outcome measures** | **Secondary outcome measures** |
| 1. Comparison of artesunate and quinine in the treatment of severe Plasmodium falciparummalaria at Kassala hospital, Sudan^1^ | Children and adults  Sample size: 94 | Open-label  Phase II  RCT | Intravenous artesunate at 2.4 mg/kg at 0, 12, and 24 hours, then daily  Or intravenous quinine at a 20 mg/kg loading dose, then 10 mg/kg three times a day | Fever and parasite clearance and coma resolution time | *Not provided* |
| 2. Phase II trial on the use of Dextran 70 or starch for supportive therapy in Kenyan children with severe malaria^2^ | Children of >6 months  Sample size: 160 | Open-label,  Phase II  RCT | Dextran 70 and hydroxyethyl starch | Resolution of shock over 8 hrs | - Resolution of acidosis - In-hospital mortality - Adverse events (allergic reactions, pulmonary edema, and neurologic sequelae) |
| 3. Intravenous artesunate plus artemisinin based Combination Therapy (ACT) or intravenous quinine plus ACT for treatment of severe malaria in Ugandan children: a randomized controlled clinical trial^3^ | Children aged 6 months and above  Sample size: 300 | Single-blind  Phase III  RCT | Intravenous artesunate (AS) or intravenous quinine (QNN) followed by oral artemisinin based combination therapy (ACT) | Parasitological treatment failure unadjusted by genotyping classified as parasitemia detected by thick blood smear during follow-up | - Parasitological treatment failure adjusted by genotyping classified as positive PCR on any follow-up day categorised as reinfection or recrudescence. - Adverse events were defined as any medical occurrence post study drug administration. They were graded as mild, moderate, severe and life threatening and their relationship to the study drug was classified as unrelated, possibly, probably or definitely related to study drug |
| 4. Artesunate versus quinine in the treatment of severe falciparum malaria in African children (AQUAMAT): an open-label, randomised trial^4^ | Children (<15 years)  Sample size: 5425 | Multicentre  Open-label  RCT  Phase III | Parenteral artesunate or parenteral quinine | In-hospital mortality compared between treatments on an intention-to-treat basis | - Severe neurological complications (assessed at 28 days, range 3–8 weeks) and a combined outcome measure of death and severe persistent neurological sequelae |
| 5. Comparison of artesunate and quinine in the treatment of Sudanese children with severe Plasmodium falciparum malaria^5^ | Children  Sample size:66 | Open-label  RCT  Phase II | Parenteral artesunate or parenteral quinine | Fever clearance time  Parasite clearance time  Coma resolution time for those in a coma  Adverse events |  |
| 6. Timing of enteral feeding in cerebral malaria in resource-poor settings: a randomized trial^6^ | Adults and children ≥2 years  Sample size:56 | RCT  Phase II | ‘early feeding’ OR ‘late feeding’  1. Enteral feeding upon admission through the NG tube  2. No feeding until able to take oral food or maximum until 60 hours after admission, followed by enteral feeding | Incidence of aspiration pneumonia  Hypoglycaemia (<2.8 mmol/L) Coma recovery time (defined as the time to a Glasgow Coma Scale of 15/15 or BCS 5/5 in preverbal children) | - Sepsis Incidence - Time to sit independently - Time to speak - Time to eat independently - Total duration (days) of admission in the hospital - Survival (In-hospital mortality) |
| 7. Levamisole hydrochloride as adjunctive therapy in severe falciparum malaria with high parasitaemia^7^ | Adults aged ≥16 years  Sample size:56 | Open-label Phase II  RCT | levamisole hydrochloride or no adjuvant to antimalarial treatment with intravenous Artesunate | Sequential assessment of peripheral blood parasitaemia and parasite stages. If sequestration is indeed reduced by levamisole, an initial increase in peripheral parasitaemia, and an increase in the number of late stages in the peripheral blood smear can be expected. | - Microvascular flow measured using orthogonal polarisation spectral imaging - Lactate clearance time |
| 8. Effect of vitamin A adjunct therapy for cerebral malaria in children admitted to Mulago hospital: a randomized controlled trial^8^ | children aged 6–59 months  Sample size:142 | Double-blind  placebo controlled  Phase II  RCT | Intravenous quinine dihydrochloride  OR  Quinine sulphate | Coma recovery time  Time for convulsions to stop  Parasite and fever clearance | - Overall mortality and time taken to start oral feed |
| 9. Safety and Preliminary Efficacy of L-arginine in Severe Falciparum Malaria (ARGISM)^9^ | Patients aged 18-60 years  Sample size: 8 | RCT  Phase II  Parallel Assignment  Triple Masking: (Participant, Care Provider, Outcomes Assessor) | Intervention: Drug: L-arginine hydrochloride  OR  Placebo Comparator:  Normal saline infusion | Improvement in endothelial function and lactate clearance | - - Safety: Clinical and biochemical measures. [ Time Frame: During and after infusion. In those receiving L-arginine, biochemical and hemodynamic measures at the completion of infusion will also be compared with measures at the start of infusion. ] - Change in endothelial function in each arginine infusion regimen vs saline placebo combined [ Time Frame: 1 hour response and end of infusion response ] - Paired change in endothelial function [ Time Frame: paired comparison of post-vs pre-infusion values, overall, and in each arginine infusion regimen ] - Lactate clearance for each infusion regimen [ Time Frame: Time for lactate to return to upper limit of normal ] - Lactate: pyruvate ratio [ Time Frame: area under curve/time to normal ] - Fever clearance time [ Time Frame: Fever clearance time ] - parasite clearance time [ Time Frame: parasite clearance time ] - Change in L-arginine concentration [ Time Frame: at 1 and 8 hours ] - Improvement in microvascular obstruction (OPS) [ Time Frame: at 1 and 8 hours ] - Tissue oxygen consumption and delivery (NIRS) [ Time Frame: one and eight hours ] - change in exhaled NO [ Time Frame: one and eight hours ] - improvement in endothelial activation (decrease in angiopoietin-2 concentrations) [ Time Frame: area under curve ] - improvement in RHPAT among those with baseline dysfunction (RHPAT<1.67) [ Time Frame: 8 hours ] |
| 10. Effect of Paracetamol on Kidney Function in Severe Malaria^10^  Identifier*:* NCT04251351  First posted: 31/Jan/2020 | Children aged 1 to 14 Years  Sample size:460 | Open Label Phase III  RCT  Parallel | Experimental:  Paracetamol  Other names:  Acetyl-Para-Aminophenol (APAP)  Acetaminophen  Sham Comparator: Arm 2 :Mechanical antipyresis (i.e. loose clothing, tepid sponging, fanning and cooling blanket) | Acute kidney injury (AKI) or death among patients enrolled without AKI (Composite outcome) [ Time Frame: during first 7 days of enrolment ]  Composite outcome of development of AKI (defined as creatinine ≥26.5 µmol/L or ≥1.5x baseline), or death.  Acute kidney injury (AKI) progression or death among patients enrolled with AKI (Composite outcome) [ Time Frame: during first 7 days of enrolment ]  Composite outcome of worsening of AKI (defined as creatinine ≥2x baseline, or ≥3x baseline, or initiation of RRT or eGFR <35 ml/min/ 1.73 m2) or death. | - Number of patients with serious adverse events [ Time Frame: during the first 5 days from enrolment ] - Number of patients with serious adverse events (mortality and/or hepatotoxicity, as defined by Hy's Law). - Proportion of patients who develop Major Adverse Kidney Events (MAKE) composite [ Time Frame: 90 days ]   Major Adverse Kidney Events (MAKE) composite, defined as ≥ 1 efficacy renal endpoints: (i) death, (ii) need for renal replacement therapy, (iii)≥ 50% reduction in eGFR from baseline to 90 days.   - Fever clearance time [ Time Frame: 6-hourly temperature assessments during first 7 days from enrolment ]   Time taken for aural temperature: (i) to fall < 37.5°C (FCT-A), and (ii) to fall < 37.5°C and remain there for >24 h (FCT-B)   - Coma recovery [ Time Frame: 6-hourly GCS/BCS assessments during first 7 days from enrolment ] - Time until Glasgow Coma Score (GCS) return to 15 (or Blantyre Coma Score (BCS) return to 5 in preverbal children) - Longitudinal change in renal function [ Time Frame: During the first 3 days from enrolment ]   As measured by creatinine concentration (umol/L)   - Longitudinal change in markers of hemolysis [ Time Frame: during the first 3 days from enrolment ]   As measured by cell-free haemoglobin (ug/mL), haemopexin (ug/mL), haptoglobin (ug/mL), haem (uM), F2-isoprostane (pg/mL) and isofurans (pg/mL) concentrations   - Longitudinal change of endothelial activation [ Time Frame: during the first 3 days from enrolment ]   As measured by concentrations of angiopoietin-Tie2 pathway markers (i.e. Ang-1, Ang-2, sTie2, sTie1)   - Longitudinal change of immune activation [ Time Frame: during the first 3 days from enrolment ] - As measured by soluble triggering receptor expressed on myeloid cells concentration (sTREM-1; pg/mL) - Longitudinal change of AKI biomarker [ Time Frame: during the first 3 days from enrolment ]   As measured by cystatin-C concentration (Cys-C; ug/mL)   - Parasite (parasites/ul) clearance [Time Frame: 12-hourly parasitemia assessments during first 7 days from enrolment] as measured by time until two consecutive negative smears (hours), and by rate using the parasite clearance estimator to determine slope half-life (hours) from 12-hourly parasite counts. - Exploratory analysis with sex [ Time Frame: During first 7 days from enrolment ] - Primary efficacy analyses will be analysed using a logistic regression model to obtain odds ratios, comparing the odds of a combined endpoint of kidney function deterioration or death between treatment groups. A multivariable model including an interaction term (sex and treatment) will be assessed in the primary analyses to explore potential differences between males and females. - Pharmacokinetic properties [ Time Frame: during the first 24 hours from enrolment ] - Population pharmacokinetic model (relative bioavailability, mean transit absorption time (hours), apparent oral elimination clearance (L/hours), apparent volume of distribution (L) - Pharmacokinetic properties [ Time Frame: during the first 24 hours from enrolment ] - Peak plasma concentration (Cmax; mg/L) - Pharmacokinetic properties [ Time Frame: during the first 24 hours from enrolment ] - Time to peak plasma concentration (Tmax; hours) - Pharmacokinetic properties [ Time Frame: during the first 24 hours from enrolment ] - Terminal elimination (t1/2; hours) - Pharmacokinetic properties [ Time Frame: during the first 24 hours from enrolment ] - Area under the plasma drug concentration-time curve (AUC0-24; mg×h×L-1) - Pharmacodynamic relationships [ Time Frame: during first 7 days from enrolment ] - Pharmacodynamic effects on creatinine concentration (mol/L) - Pharmacodynamic relationships [ Time Frame: during first 7 days from enrolment ] - Pharmacodynamic effects on liver toxicity, as measured by AST and ALT (U/L) - Pharmacodynamic relationships [ Time Frame: during first 7 days from enrolment ] - Pharmacodynamic effects on temperature (Celsius) - Pharmacodynamic relationships [ Time Frame: during first 7 days from enrolment ] - Pharmacodynamic effects on parasitemia, as measured by parasites/ul and slope half-life - Pharmacodynamic relationships [ Time Frame: during first 7 days from enrolment ] - Pharmacodynamic effects on GCS (or BCS in pre-verbal children) |
| 11. Treating Brain Swelling in Paediatric Cerebral Malaria (TBS)^11^  Identifier: NCT03300648  First Posted: 3/Oct/2017 | Children aged 6 Months to 12 Years  Sample size:261 | Randomized  Parallel Assignment  Single Masking(Outcomes Assessor)  Phase III | Three study arms:   - No Intervention: Usual care - Experimental: Mechanical ventilation, Intubation and mechanical ventilation for a maximum of 7 days - Drug: Hypertonic saline Intravenous 3 percent hypertonic saline for a maximum of 7 days | Mortality  Time Frame: Within 7 days of randomization | - Neurodevelopmental disability [Time Frame: 1 year] - Presence and severity of neurodevelopmental disability in survivors |
| 12. Effect of Paracetamol on Renal Function in Plasmodium Knowlesi Malaria (PACKNOW)^12^  Identifier: NCT03056391  First Posted: 17/Feb/2017 | Patients aged ≥5 years  Sample size:360 | RCT  Phase III  Open Label | Experimental: Paracetamol plus IV artesunate or oral artemether/lumefantrine  No Intervention: No Paracetamol plus IV artesunate or oral artemether/lumefantrine. | Effect of Paracetamol on kidney function [ Time Frame: 72 hours ]  Change in creatinine concentration (umol/L) at 72 hours from enrolment in patients receiving regularly-dosed paracetamol compared to those not receiving regular paracetamol, stratified by the level of intravascular haemolysis (cell-free haemoglobin) | - Longitudinal change in creatinine [ Time Frame: 72 hours ]   Longitudinal change in creatinine, as measured by the area under the creatinine-time curve, with creatinine measured 12 hourly from enrolment to 72 hours; and the effect of enrolment cell-free haemoglobin on longitudinal change in creatinine   - Change in creatinine in severe malaria [ Time Frame: 72 hours ]   Change in creatinine at 72 hours and longitudinal change in creatinine over 72 hours, including the effect of enrolment CFHb, in patients with severe knowlesi malaria.   - Development of AKI [ Time Frame: 72 hours ]   Development of AKI over 72 hours: i) an absolute increase in serum creatinine of >26.5 umol/L from enrolment creatinine; ii) a percentage increase in serum creatinine of >50% from enrolment; iii) post-enrolment onset of oliguria of less than 0.5ml/kg/hour for more than 6 hours; iv) 24 hour urine output of <400ml after rehydration and urinary obstruction excluded. AKI on enrolment will also be described by the Kidney Disease Improving Global Outcomes (KDIGO) criteria (with baseline creatinine estimated using the MDRD equation).   - Duration of AKI [ Time Frame: 28 days ]   Length of time elapsed until serum creatinine returns to normal (estimated using MDRD equation) in the absence of renal replacement therapy in those with AKI on enrolment and those that develop AKI after enrolment.   - Longitudinal changes in haemolysis: plasma cell-free haemoglobin [ Time Frame: 72 hours ]   Longitudinal changes in plasma cell-free haemoglobin over 72 hours.   - Longitudinal changes in haemolysis: plasma cell-free haem [ Time Frame: 72 hours ]   Longitudinal changes in plasma cell-free haem over 72 hours.   - Longitudinal changes in haemolysis: haem-to-protein cross-links [ Time Frame: 72 hours ]   Longitudinal changes in haem-to-protein cross-links over 72 hours.   - Longitudinal changes in markers of oxidative stress: F2-IsoP [ Time Frame: 72 hours ]   Longitudinal changes in plasma F2-isoprostanes [F2-IsoP] over 72 hours.   - Longitudinal changes in markers of oxidative stress: IsoF [ Time Frame: 72 hours ]   Longitudinal changes in plasma isofurans [IsoF]) over 72 hours.   - Longitudinal changes in F2-IsoPs according to G6PD enzyme activity [ Time Frame: 72 hours ]   Longitudinal changes in F2-IsoPs according to G6PD enzyme activity, assessed qualitatively by fluorescent spot test.   - Longitudinal changes in IsoFs according to G6PD enzyme activity [ Time Frame: 72 hours ]   Longitudinal changes in IsoFs and CFHb according to G6PD enzyme activity, assessed qualitatively by fluorescent spot test.   - Longitudinal changes in CFHb according to G6PD enzyme activity [ Time Frame: 72 hours ]   Longitudinal changes in CFHb according to G6PD enzyme activity, assessed qualitatively by fluorescent spot test.   - Longitudinal changes in F2-IsoPs according to G6PD genotype [ Time Frame: 72 hours ]   Longitudinal changes in F2-IsoPs according to G6PD genotype   - Longitudinal changes in IsoFs according to G6PD genotype [ Time Frame: 72 hours ]   Longitudinal changes in IsoFs according to G6PD genotype   - Longitudinal changes in CFHb according to G6PD genotype [ Time Frame: 72 hours ]   Longitudinal changes in CFHb according to G6PD genotype   - Population pharmacokinetics of paracetamol: Cmax [ Time Frame: 72 hours ]   Peak plasma concentration (Cmax)   - Population pharmacokinetics of paracetamol: Tmax [ Time Frame: 72 hours ]   Time to peak plasma concentration (Tmax)   - Population pharmacokinetics of paracetamol: AUC [ Time Frame: 72 hours ]   Area under the plasma drug concentration-time curve (AUC)   - Population pharmacodynamics of paracetamol [ Time Frame: 72 hours ]   Paracetamol dose-response curve   - Fever clearance time [ Time Frame: 72 hours ]   Defined as the time taken for the aural temperature to fall below 37.5°C, and the time taken for the temperature to fall below 37.5°C and remain there for at least 24hours   - Fever duration [ Time Frame: 72 hours ]   Defined as the duration in hours that an individual's temperature is above 37.5°C   - Area above the fever versus time curve (AUC-T°) [ Time Frame: 72 hours ]   Area above the 37.5°C temperature versus time curve (AUC-T°) within first 24 hours of treatment.   - Parasite clearance time (hours) [ Time Frame: 72 hours ]   Parasite clearance time, defined as (i) the time from commencement of antimalarial treatment to the first of 2 consecutive negative blood films, with blood films assessed by microscopy every 6 hours for the presence of asexual parasitaemia, and (ii) the linear portion of the slope of the log-parasitemia versus time relationship.   - Blood and urine biomarkers of pre-renal and renal injury [ Time Frame: 72 hours ]   Neutrophil gelatinase-associated lipocalcin (NGAL), kidney injury molecule (KIM), urinalysis, urine microscopy, urine electrolytes, and urine creatinine.   - Longitudinal urine colour [ Time Frame: 72 hours ]   Longitudinal urine colour (assessed by standardized urine colour charts). The proportion of patients with enrolment urine pH less than 6 together with a urine color of 6 or greater who develop AKI will be compared between groups.   - Longitudinal urine pH [ Time Frame: 72 hours ]   Longitudinal urinalysis dipstick test-strip: urine pH. The proportion of patients with enrolment urine pH less than 6 together with a urine color of 6 or greater who develop AKI will be compared between groups.   - Longitudinal urine specific gravity [ Time Frame: 72 hours ]   Longitudinal urinalysis dipstick test-strip: urine specific gravity   - Longitudinal urine haemoglobin [ Time Frame: 72 hours ]   Longitudinal urinalysis dipstick test-strip: urine haemoglobin   - Change in creatinine (umol/L) between therapeutic concentrations of paracetamol vs those with absent or low. [ Time Frame: 72 hours ]   Change in creatinine at 72 hours and longitudinal change in creatinine over 72 hours in patients with therapeutic concentrations of paracetamol, compared to patients with absent or low concentrations of paracetamol   - Number of participants with treatment-related adverse events as assessed by CTCAE v4.0 [ Time Frame: 28 days ]   Reporting of any unfavourable and unintended sign (including an abnormal laboratory finding), symptom, or disease temporally associated with paracetamol administration   - Longitudinal red cell deformability [ Time Frame: 72 hours ]   Longitudinal red cell deformability, as measured by laser-assisted optical rotational red cell analyser (LORCA) elongation index.   - Longitudinal changes in markers of endothelial dysfunction [ Time Frame: 72 hours ]   Longitudinal changes in markers of weibel palade body exocytosis including angiopoietin-2 |
| 13. Rosiglitazone Adjunctive Therapy for Severe Malaria in Children (ROSI)^13^  Identifier: NCT02694874  First Posted : 01/March/2016 | Children aged 1-12 years  Sample size:210 | RCT  Masking: Quadruple (Participant, Care Provider, Investigator, Outcomes Assessor) Phase III | Experimental: Rosiglitazone  Comparator:  Placebo | Change in serum Ang-2 levels in the first 96 hours of hospital admission. [ Time Frame: first 96 hours of hospital admission. ]  We will assess the effect of the intervention (vs. placebo) on Ang-2 levels as a biomarker of severe disease in severe malaria | 1. Time to clinical recovery [Time Frame: up to 96 hours after hospital admission]. Time to recovery including: 2. Time to fever resolution for at least 24h. Temperature measurements will be taken at admission and every 4h for the first 4 days, and then every 12h until 2 normal results (<37.5oC) are reported. 3. Time to sit unsupported 4. Time to hospital discharge 5. Time to parasitological recovery [ Time Frame: up to 96 hours after hospital admission ]   Time to parasitological recovery: Time (in hours) to clearance of parasitemia from the blood (both 50% and 90% decrease from admission baseline value). Parasitemia will be quantified at admission and every 6h, for 4 days or until 2 negative readings are reported.   1. Mortality [ Time Frame: first 48h post-hospital admission and at 14 days post-hospital admission ]   Mortality in the first 48h post-hospital admission and at 14 days post-hospital admission   1. Blood lactate levels, assessed at admission, every 12h for 24 hours then daily for Blood lactate levels [ Time Frame: Assessed at admission, every 12h for 24 hours then daily for 4 days, and once on day 14 and 6 month follow ups ].   Blood lactate levels, assessed at admission, every 12h for 24 hours then daily for 4 days, and once on day 14 and 6 month follow ups   1. Change in levels of biomarkers of host response [ Time Frame: at admission, every 12h for 24 hours then daily for 4 days, and once on day 14 and 6 month follow ups ]   Change in levels of biomarkers of host response at admission, every 12h for 24 hours then daily for 4 days, and once on day 14 and 6 month follow ups   1. Blood glucose levels [ Time Frame: up to 96 hours after hospital admission ]   Blood glucose levels assessed at admission and every 6h for the first 48h, and then every 24h for following 2 days   1. Cardiac effects [ Time Frame: from baseline to 24h, and day 4 ]   Monitor for cardiac effects by conducting ECG at baseline, at 24h (immediately before third doses of rosiglitazone and artesunate treatment are administered) and at the end of rosiglitazone treatment (day 4). Main outcome of interest will be changes in QTc from baseline to the two different time points.   1. Biochemical and hematological parameters [ Time Frame: up to 96 hours after hospital admission ]   Biochemical and hematological parameters including: AST, ALT, creatinine, complete blood count (e.g. hemoglobin, WBC and differential, hematocrit, platelet count) will be assessed at admission and every 24h until day 4   1. AE/SAE [ Time Frame: up to day 14 after hospital admission ]   AE/SAE monitored using the paediatric toxicity tables modified from the US National Institutes of Allergy and Infectious Diseases   1. Neurocognitive outcomes [ Time Frame: From baseline to 6 months post discharge, and 18 months post discharge ]   Participants with Adverse Events that Are Related and unrelated to Treatment by a variety of standard neurocognitive tests |
| 14. A Trial of the Efficacy of Artesunate and Three Quinine Regimens in the Treatment of Severe Malaria in Children at the Ebolowa Regional Hospital – Cameroon^14^  Identifier: NCT02563704  First Posted: 30/Sept/2015 | Children aged 3 Months to 15 Years  Sample size:238 | Open Label RCT  Phase III | Artesunate  Quinine | - Fever clearance time [ Time Frame: Time (in hours) from the onset of treatment till rectal temperature went down to 37.5°C for at least 24 hour ] - Coma recovery time [ Time Frame: Time (in hours) from the onset of treatment till the participant was fully conscious with a BCS of 5 or GCS of 15 for an average of 24 hours ] - Time to sit unsupported [ Time Frame: Time (in hours) from the onset of treatment till when the participant could sit unsupported for an average of 24 hours if the participant was unable to do so on admission ] - Time to eat and drink [ Time Frame: Time (in hours) from the onset of treatment till when the participant could eat and drink for an average of 24 hours if the participant was unable to do so on admission ] - Parasite clearance time [ Time Frame: Time (in hours) from the onset of treatment to the time of the first of two successive negative blood smear through hospital discharge, an average of one week ] - Parasite reduction rate 24 hours after onset of treatment [ Time Frame: 24 hours from onset of treatment ] | *Not Provided* |
| 15. Bio-availability of Rectal Artesunate in Children With Severe Falciparum Malaria (REACH)^15^  Identifier: NCT02492178  First Posted:  08/Jul/2015 | Child, Adult, Older Adult Weight ≥6 kilograms and ≤ 34 kilograms  Sample size:82 | Open Label RCT  Phase II | Intrarectal Artesunate  Intravenous Artesunate  Intravenous quinine | - Pharmacokinetics profile of rectal artesunate [ Time Frame: 24 hours ] - The pharmacokinetics profile of rectal artesunate (the study drug), consisting of: area under the concentration-time curve; terminal elimination half-life; elimination clearance; apparent volume of distribution will be measured and compared to the pharmacokinetic profile of intravenous artesunate (the comparator). | *Not Provided* |
| 16. A Safety and Feasibility Study of Enteral LVT vs. Standard of Care for Seizure Control in Pediatric CM (LVT2)^16^  Identifier: NCT01982812  First Posted: 13/Nov/2013 | Children aged 24 to 83 Months  Sample size:44 | RCT  Phase II  Open Label | Drug: Oral Levetiracetam Drug: Standard antiepileptic drug (AED) | 1. Minutes With Seizure on EEG [ Time Frame: 72 hours ]   Comparing LVT to standard AED the number of minutes spent in seizure per EEG in the 72 hours after treatment allocation. | - Required Additional AED [ Time Frame: 7 days ]   Additional AEDs required (including for breakthrough seizures in LVT group) during admission for seizure control (yes/no)   - Mean Time From Admission to BCS >/= 4 [ Time Frame: 7 days ]   The mean time in hours from admission until the subject reaches Blantyre Coma Scale of greater than or equal to 4. Participants who died are excluded from this analysis.   - Sequelae [ Time Frame: 7 days ]   Neurologic outcome in 3 categories:   1. Neurologically intact at discharge 2. Neurologic sequelae at discharge--specifically new sensory or motor deficits, ongoing seizures, or behavioral abnormalities based upon a physician examination at discharge 3. Died during admission, never discharged |
| 17. Intravenous Artesunate and Malaria (IVAS)^17^  Identifier: NCT01805232  First Posted: 06/Mar/2013 | Patients aged between 1 to 80 Years  Sample size:80 | Open Label RCT  Phase II | Artesunate  Or  Quinine | Clearance of the parasite and fever [Time Frame: 3 days]  To compare the parasite clearance time and fever clearance time between the two groups | *Not Provided* |
| 18. Acute vs. Delayed Iron: Effect on Red Cell Iron Incorporation in Severe Malaria^18^  Identifier: NCT01754701  First Posted:21/Dec/2012 | Children aged 6 to 59 months  Sample size:100 | Open Label RCT  Phase II | Dietary Supplement: Immediate iron  Or  Delayed iron | Percent red blood cell iron incorporation on Day 0 in children in the immediate group vs  percent red blood cell iron incorporation on Day 28 in children in the delayed group [ Time Frame: 56 days ] | Hematological recovery in the immediate vs. delayed groups on Day 56 [ Time Frame: 56 days ] |
| 19. Paracetamol Effect on Oxidative Stress and Renal Function in Severe Malaria^19^  Identifier: NCT01641289  First Posted: 16/Jul/2012 | 12 Years and older (Child, Adult, Older Adult)  Sample size:62 | Open Label Phase II  RCT | Experimental: Paracetamol plus intravenous Artesunate  Active Comparator:  No paracetamol plus Intravenous Artesunate | Effect of paracetamol concentrations [ Time Frame: 72 hours ]  Compare the effect of therapeutic paracetamol concentrations compared with absent or low paracetamol concentration on renal function, peak creatinine levels or trough creatinine clearance, defined as the change at 72 hours compared to baseline, in patients with severe and moderately severe falciparum malaria stratified by the level of intravascular haemolysis (cell-free haemoglobin). | - Compare treatment arm with control arm with respect to duration of Acute Kidney Injury (AKI) and development of AKI. [ Time Frame: 14 days ]   Duration of AKI will be defined as the length of time elapsed until serum creatinine returns to normal (<1mg/dL) in the absence of renal replacement therapy. Development of AKI will be assessed using the Acute Kidney Injury Network (AKIN) criteria, and by a creatinine increase of >= 0.5mg/dl or 25%. Plasma paracetamol concentration will be measured daily by liquid chromatography-mass spectrometer (LC-MS/MS).   - Compare between groups correlations between oxidative stress, cell-free hemoglobin and renal function [ Time Frame: 3 days ]   Urine and plasma F2-isoprostanes and isofurans will be measured by gas chromatography-mass spectrometry. Cell-free haemoglobin measured as plasma concentration by enzyme linked immunosorbent assay (ELISA) on admission then daily for 72hours. Cell free haem measured in plasma using a chromogenic assay on admission then daily for 72hours. Haem-to-protein cross-links measured by high performance liquid chromatography (HPLC) on admission and daily for 72hours.   - Assessment of Blackwater fever and association with renal function [ Time Frame: 7 days ]   Blackwater fever assessed using a standardized urine colour chart and urine haemoglobin every 6 hours until clinical recovery between groups.   - Mortality and hemodialysis trends [ Time Frame: 4 weeks ]   To compare mortality and hemodialysis trends between groups and evaluate if they correlate with level of oxidative stress, cell free haemoglobin and renal function.   - Host factors of Intravascular Haemolysis [ Time Frame: 4 weeks ]   Intravascular haemolysis according to G6PD status.   - Fever clearance time [ Time Frame: 7 days ]   Compared fever clearance time defined as the time taken for the tympanic temperature to fall below 37.5°C and remain there for at least 24hours); Fever time defined as the duration in hours of an individuals temperature above 37.5°C; Area above the 37.5°C temperature versus time curve (AUC-T°) within first 24hours of treatment. Aural temperature will be measured every 6 hours until fever clearance.   - Parasite clearance time [ Time Frame: 7 days ]   Parasite clearance time assessed by microscopy of peripheral blood films will be assessed every 6hours for the presence of asexual parasitaemia until negative on 2 consecutive blood films. Parasite half lives and clearance time will be compared between groups. Parasites will also be staged to assess if sequestration is inhibited due to temperature reduction.   - Parasite sequestration [ Time Frame: 7 days ]   Parasite sequestration assessed by capillary flow in the rectal microcirculation using Orthogonal Polarization Spectral (OPS) imaging will be compared between groups.   - Assessment of Acute Kidney Injury [ Time Frame: 7 days ]   Evaluation of pre-renal and acute tubular necrosis assessed by blood and urine biomarkers of pre-renal and renal injury including neutrophil gelatinase-associated lipocalcin (NGAL) and kidney injury molecule (KIM).   - Urine scoring of dehydration and haemolysis [ Time Frame: 72 hours ]   Urine colour will be assessed by standardized urine colour charts. Urine colour will be correlated with urine specific gravity, urine osmolality, urine haemoglobin and creatinine clearance.   - Safety assessment [ Time Frame: 6 weeks ]   Safety assessed by the number of patients with serious adverse events (SAEs) and by changes from baseline in vital signs and laboratory measurements.   - Assess the antimalarial drug sensitivity of patients treated with paracetamol [ Time Frame: 72 hours ]   Preliminary in vitro studies suggest that paracetamol potentiates the anti-parasitic effect of artesunate as lower 50% inhibitory concentration of artesunate is observed when paracetamol is added to parasites in culture. We will investigate whether this effect is dependent on the infecting P. falciparum parasite strain   - Paracetamol pharmacokinetics [ Time Frame: 72 hours ]   Pharmacokinetic modelling of oral paracetamol in severe and moderately severe malaria   - Paracetamol pharmacodynamics [ Time Frame: 72 hours ]   Pharmacodynamics on variables including temperature and parasitemia.   - Area under the plasma concentration versus time curve (AUC) [ Time Frame: 72 hours ]   for paracetamol   - The maximum concentration (Cmax) [ Time Frame: 72 hours ]   for paracetamol |
| 20. Evaluation of the Efficacy and Safety of Inhaled Nitric Oxide As Adjunctive Treatment for Cerebral Malaria in Children^20^  Identifier: NCT01388842  First Posted : 07/Jul/2011 | Children aged 2 Months to 12 Years  Sample size:92 | Open Label  Phase II  RCT | Drug: inhaled nitric oxide Drug: Placebo | Angiopoietin 1 (Ang-1) [ Time Frame: 48 hours ]  Increase in Ang-1 between inclusion and 48 hours of combined therapy (iNO or placebo plus antimalarial chemotherapy) | - Mortality [ Time Frame: 48 hours ]   Reduction in mortality at 48 hours   - Coma score [ Time Frame: 48 hours ]   Normalisation of coma score (Blantyre coma scale)   - Retinopathy [ Time Frame: every 6 hours ]   Normalisation of malaria retinopathy measured by indirect fundoscopy   - Tone [ Time Frame: 48 hours ]   Improvement of posture and tone   - Measure of occurrence of neurological sequelae in children [ Time Frame: months 1, 3 and 6 ]   Reduction of incidence of neurological sequelae, including motor dysfunction, behavioural disorders, hearing, speech and sight disorders and seizure disorders.   - Vital signs [ Time Frame: every 6 hours ]   Improvement of vital signs: Systolic and diastolic blood pressure, pulse rate, temperature   - Oxygen saturation [ Time Frame: every 6 hours ]   Both Hb Oxygen saturation (SpO2) and total MetHb levels continuously measured by pulse oximetry (Rascal Model 7, Massimo Corp.) |
| 21. Inhaled Nitric Oxide for the Adjunctive Therapy of Severe Malaria: a Randomized Controlled Trial^21^  Identifier: NCT01255215  First Posted: 07Dec/2010 | Children aged 1-10 years  Sample size:180 | Phase I  Phase II  RCT  Quadruple masking (Participant, Care Provider, Investigator, Outcomes Assessor) | Intervention: Inhaled Nitric Oxide  Or  Placebo Comparator: Room air | Change in serum angiopoietin-2 level [ Time Frame: Admission through 72 hours ] | - Mortality [ Time Frame: 48 hours and 14 days after admission ] - Time to hospital discharge [ Time Frame: From admission to approximately 72 hours ] - Time to parasite clearance. [ Time Frame: From admission to approximately 72 hours ] - Biomarkers and genetic determinants of endothelial activation, inflammation and coagulopathy, to be determined. [ Time Frame: From admission to approximately 72 hours ] |
| 22. The pharmacology of azithromycin in severe malaria bacterial co-infection in African children^22^  Identifier: ISRCTN49726849  Registered:24/10/2017 | Children aged six months to 12 years old who are admitted to the hospital with malaria  Sample size:105 | Randomised parallel trial  Phase I/II | Children are randomised (1:1:1) to compare three doses of azithromycin: 10, 15 and 20 mg/kg (based on weight-bands) taken orally once daily over five days in order to optimize dose and study pharmacokinetics and their relation with treatment outcome. | Sepsis markers are measured using the c-reactive protein tests using blood samples at baseline to 72 hours (continuous) and microbiological cure (7-day). | - Mortality is measured using dedicated case report forms at clinical visit or telephone interviews at 48 days and 90 days - Length of hospital stay is measured using dedicated case report forms - Re-hospitalisation measured using parental interview using case report forms at follow up visits on Day 7, Day 28 and Day 90 - 4. Adverse events is measured using dedicated serious adverse event forms during hospital admission and follow up visits on Day 7, Day 28 and Day 90 |
| 23. Aggressive Antipyretics for Fever Reduction in CNS Malaria^23^  First posted: 27/03/2018  Identifier: PACTR201804003255157 | Children aged 2 Years to 11 Years  Sample size: 284 | Phase II  RCT  Double-Blinded | Acetaminophen  Or  Ibuprofen  Or placebo | Mean maximum temperature (TMAX) [ Time Frame: 72 hours ]  TMAX defined as highest temperature during study duration, 72 hours, in degrees Celsius recorded by continuous temp monitor. Continuous temp monitors not MRI compatible. | - Seizures   Seizures detected clinically or on daily EEG during the study duration (72 hours)   - Parasite burden   Based upon HRP2 levels and quantitative blood film Q6 hourly until aparasitemic on thick blood smear, 72 hours. |
| 24. Intramuscular artesunate for Severe Malaria in African Children: A Multicenter Randomized Controlled Trial^24^  First posted: 07/02/2011  Identifier: PACTR201102000277177 | Children aged 6 months to 10 Year  Sample size: 1047 | Phase II  Open-label  Non-inferiority RCT | 3-dose versus 5-dose regimen of intravenous or intramuscular application of artesunate | The proportion of patients with parasite clearance (>99% reduction from Baseline) at 24hrs after initiation of study drug | - Pharmacokinetic sample collection for subgroup of 300 patients - Non-invasive oto-acoustic test - LODS - Genetic polymorphisms - In vitro drug sensitivity (only in Gabon) |
| 25. Brain Swelling and Mannitol Therapy in Adult Cerebral Malaria, A Randomized Trial^25^ | Adult patients aged ≥16 years  Sample  Size: 61 | RCT  Phase II  Open-label  Single-blind | Adjunctive treatment with intravenous  mannitol  OR no adjunctive therapy | Coma recovery time of 24 hours | Mortality |
| 26. Randomized controlled trial of artesunate or artemether in Vietnamese adults with severe falciparum malaria^26^ | Patients aged ≥14 years  Sample size:370 | RCT  Phase III  double blind | Intramuscular  artesunate  OR  intramuscular artemether | Mortality rate | - Convulsions - Required blood transfusion - Renal impairment - Renal failure - Required dialysis - Hypoglycaemia - Spontaneous bleeding - Shock - Concomitant infection - Jaundice - Pulmonary oedema |
| 27. Ursodeoxycholic acid and artesunate in the treatment of severe falciparum malaria patients with jaundice^27^ | Patients aged ≥15 years  Sample size:74 | Phase II  RCT | Oral Ursodeoxycholic acid  OR placebo  In addition to artesunate | Improvement in liver tests as measured by the mean change of percentage of the glutamyl transpeptidase (GGT) level after treatment (a cholestatic parameter).  For obtaining reasonably estimates for the primary end-point of  improvement in liver tests, we used the mean change of percentage  of the glutamyl transpeptidase (GGT) level after treatment (a  cholestatic parameter) as our primary parameter.  For obtaining reasonably estimates for the primary end-point of  improvement in liver tests, we used the mean change of percentage  of the glutamyl transpeptidase (GGT) level after treatment (a  cholestatic parameter) as our primary parameter. | - Parasitaemia - Adverse events |

| **Supplementary Table 3: Outcome measures and their definitions** | | | |
| --- | --- | --- | --- |
| **Outcome** | Primary or secondary outcome? | Anti-malarial or adjunctive therapy? | Definition used in publications |
| **Renal function** |  |  |  |
| Acute kidney injury (AKI) or death among patients enrolled without AKI (Composite outcome) [ Time Frame: during first 7 days of enrolment ]^10^ | Primary | Adjunctive therapy | Composite outcome of development of AKI (defined as creatinine ≥26.5 µmol/L or ≥1.5x baseline), or death at any timepoint |
| Acute kidney injury (AKI) progression or death among patients enrolled with AKI (Composite outcome) [ Time Frame: during first 7 days of enrolment ]^10^ | Primary | Adjunctive therapy | Composite outcome of worsening of AKI (defined as creatinine ≥2x baseline, or ≥3x baseline, or initiation of RRT or eGFR <35 ml/min/ 1.73 m2) or death at any timepoint |
| Proportion of patients who develop Major Adverse Kidney Events (MAKE) composite [ Time Frame: 90 days ]^10^ | Secondary | Adjunctive therapy | Major Adverse Kidney Events (MAKE) composite, defined as ≥ 1 efficacy renal endpoints: (i) death, (ii) need for renal replacement therapy, (iii)≥ 50% reduction in eGFR from baseline to 90 days |
| Longitudinal change in renal function [ Time Frame: During the first 7 days from enrolment ]^10^ | Secondary | Adjunctive therapy | Measured by creatinine concentration (umol/L) |
| Longitudinal change of AKI biomarker [ Time Frame: during the first 3 days from enrolment ]^10^ | Secondary | Adjunctive therapy | Measured by cystatin-C concentration (Cys-C; ug/mL) |
| Effect of Paracetamol on kidney function [ Time Frame: 72 hours ]^12^ | Primary | Adjunctive therapy | Change in creatinine concentration (umol/L) at 72 hours from enrolment in patients receiving regularly-dosed paracetamol compared to those not receiving regular paracetamol, stratified by the level of intravascular haemolysis (cell-free haemoglobin). |
| Longitudinal change in creatinine [ Time Frame: 72 hours ]^12^ | Secondary | Adjunctive therapy | Longitudinal change in creatinine, as measured by the area under the creatinine-time curve, with creatinine measured 12 hourly from enrolment to 72 hours; and the effect of enrolment cell-free haemoglobin on longitudinal change in creatinine |
| Change in creatinine in severe malaria [ Time Frame: 72 hours ]^12^ | Secondary | Adjunctive therapy | Change in creatinine at 72 hours and longitudinal change in creatinine over 72 hours, including the effect of enrolment CFHb, in patients with severe knowlesi malaria |
| Development of AKI [ Time Frame: 72 hours ]^12^ | Secondary | Adjunctive therapy | Development of AKI over 72 hours: i) an absolute increase in serum creatinine of >26.5 umol/L from enrolment creatinine; ii) a percentage increase in serum creatinine of >50% from enrolment; iii) post-enrolment onset of oliguria of less than 0.5ml/kg/hour for more than 6 hours; iv) 24 hour urine output of <400ml after rehydration and urinary obstruction excluded. AKI on enrolment will also be described by the Kidney Disease Improving Global Outcomes (KDIGO) criteria (with baseline creatinine estimated using the MDRD equation) |
| Duration of AKI [ Time Frame: 28 days ]^12^ | Secondary | Adjunctive therapy | Length of time elapsed until serum creatinine returns to normal (estimated using MDRD equation) in the absence of renal replacement therapy in those with AKI on enrolment and those that develop AKI after enrolment |
| Change in creatinine (umol/L) between therapeutic concentrations of paracetamol vs those with absent or low. [ Time Frame: 72 hours ]^12^ | Secondary | Adjunctive therapy | Change in creatinine at 72 hours and longitudinal change in creatinine over 72 hours in patients with therapeutic concentrations of paracetamol, compared to patients with absent or low concentrations of paracetamol |
| Compare treatment arm with control arm with respect to duration of Acute Kidney Injury (AKI) and development of AKI. [ Time Frame: 14 days ]^19^ | Secondary | Adjunctive therapy | Duration of AKI will be defined as the length of time elapsed until serum creatinine returns to normal (<1mg/dL) in the absence of renal replacement therapy. Development of AKI will be assessed using the Acute Kidney Injury Network (AKIN) criteria, and by a creatinine increase of >= 0.5mg/dl or 25%. Plasma paracetamol concentration will be measured daily by liquid chromatography-mass spectrometer (LC-MS/MS) |
| Compare between groups correlations between oxidative stress, cell-free hemoglobin and renal function [ Time Frame: 3 days ]^19^ | Secondary | Adjunctive therapy | Urine and plasma F2-isoprostanes and isofurans will be measured by gas chromatography-mass spectrometry. Cell-free haemoglobin measured as plasma concentration by enzyme linked immunosorbent assay (ELISA) on admission then daily for 72hours. Cell free haem measured in plasma using a chromogenic assay on admission then daily for 72hours. Haem-to-protein cross-links measured by high performance liquid chromatography (HPLC) on admission and daily for 72hours |
| Assessment of Blackwater fever and association with renal function [ Time Frame: 7 days ]^19^ | Secondary | Adjunctive therapy | Blackwater fever assessed using a standardized urine colour chart and urine haemoglobin every 6 hours until clinical recovery between groups |
| Mortality and hemodialysis trends [ Time Frame: 4 weeks ]^19^ | Secondary | Adjunctive therapy | To compare mortality and hemodialysis trends between groups and evaluate if they correlate with level of oxidative stress, cell free haemoglobin and renal function |
| Assessment of Acute Kidney Injury [ Time Frame: 7 days ]^19^ | Secondary | Adjunctive therapy | Evaluation of pre-renal and acute tubular necrosis assessed by blood and urine biomarkers of pre-renal and renal injury including neutrophil gelatinase-associated lipocalcin (NGAL) and kidney injury molecule (KIM) |
| Renal impairment  Renal failure  Required dialysis^26^ | Secondary | Anti-malarial therapy | Unspecified in the publication |
| **Temperature/fever** |  |  |  |
| Fever clearance time^1,5,14^ | Primary | Anti-malarial therapy | Fever clearance times were measured from the start of antimalarial treatment to the time at which the axillary temperature first dropped below 37.5°C and remained below 37.5°C for 24 hours |
| Fever clearance time [ Time Frame: 72 hours ]^12^ | Secondary | Adjunctive therapy | Defined as the time taken for the aural temperature to fall below 37.5°C, and the time taken for the temperature to fall below 37.5°C and remain there for at least 24hours |
| Clearance of fever [Time Frame: 3 days]^17^ | Primary | Anti-malarial therapy | Fever clearance [Time Frame: 3 days] |
| Fever clearance time [ Time Frame: 6-hourly temperature assessments during first 7 days from enrolment ]^10^ | Secondary | Adjunctive therapy | Time taken for aural temperature: (i) to fall < 37.5°C (FCT-A), and (ii) to fall < 37.5°C and remain there for >24 h (FCT-B) |
| Fever clearance time [ Time Frame: 7 days ]^19^ | Secondary | Adjunctive therapy | Compared fever clearance time defined as the time taken for the tympanic temperature to fall below 37.5°C and remain there for at least 24hours); Fever time defined as the duration in hours of an individual’s temperature above 37.5°C; Area above the 37.5°C temperature versus time curve (AUC-T°) within first 24hours of treatment. Aural temperature will be measured every 6 hours until fever clearance. |
| Fever duration [ Time Frame: 72 hours ]^12^ | Secondary | Adjunctive therapy | Defined as the duration in hours that an individual's temperature is above 37.5°C |
| Area above the fever versus time curve (AUC-T°) [ Time Frame: 72 hours ]^12^ | Secondary | Adjunctive therapy | Area above the 37.5°C temperature versus time curve (AUC-T°) within first 24 hours of treatment |
| Mean maximum temperature (TMAX) [ Time Frame: 72 hours ]^23^ | Primary | Adjunctive therapy | TMAX will be defined as the highest temperature during the study duration (72 hours) in degrees Celsius recorded by a continuous temperature monitor. |
| **Neurological** |  |  |  |
| Coma resolution time^1^ | Primary | Anti-malarial therapy | Coma recovery time (for patients with a Glasgow Coma Score (GCS) < 11 [out of 15] on admission) was measured from the start of antimalarial treatment to the time at which the score reached 15 |
| Severe neurological complications (assessed at 28 days, range 3–8 weeks) and a combined outcome measure of death and severe persistent neurological sequelae^4^ | Secondary | Anti-malarial therapy | Severe neurological complications (assessed at 28 days, range 3–8 weeks) and a combined outcome measure of death and severe persistent neurological sequelae. These sequelae were assessed in four domains: motor function, visual function, hearing, and speech, and also whether the child had developed epilepsy. |
| Coma recovery time^5^ | Primary | Anti-malarial therapy | Coma recovery time (for patients with a Glasgow Coma Score (GCS) <11 [of 15] on admission) was measured from the start of antimalarial treatment to the time at which the score reached 15. |
| Coma recovery time ^7^ | Primary | Adjunctive therapy | The coma recovery time is defined as the time until a Glasgow Coma Score (GCS) of 15/15 (BCS 5/5 in preverbal kids) |
| Coma recovery time^8^ | Primary | Anti-malarial therapy | Coma recovery time as determined by a Blantyre coma score of 5 time taken for convulsions to stop after initiating treatment and time to sit unsupported. |
| Coma recovery time^10^ | Secondary | Adjunctive therapy | Coma recovery [ Time Frame: 6-hourly GCS/BCS assessments during first 7 days from enrolment ] Time until Glasgow Coma Score (GCS) return to 15 (or Blantyre Coma Score (BCS) return to 5 in preverbal children) |
| Coma Score [ Time Frame: 48 hours ]^20^ | Secondary | Adjunctive therapy | Normalisation of coma score (Blantyre coma scale) |
| Coma recovery time^25^ | Secondary | Adjunctive therapy | Coma recovery time of 24 hours ( Coma defined as a Glasgow Coma Score (GCS) <11) |
| **Parasite clearance time** |  |  |  |
| Parasite clearance time^1,14^ | Primary | Anti-malarial therapy | Parasite clearance time was defined as the interval between the start of treatment and the time of the first of two sequential negative thick films. |
| Parasite clearance time (hours) [ Time Frame: 72 hours ]^12^ | Secondary | Adjunctive therapy | Parasite clearance time, defined as (i) the time from commencement of antimalarial treatment to the first of 2 consecutive negative blood films, with blood films assessed by microscopy every 6 hours for the presence of asexual parasitaemia, and (ii) the linear portion of the slope of the log-parasitemia versus time relationship. |
| Time to parasitological recovery [ Time Frame: up to 96 hours after hospital admission ]^13^ | Secondary | Adjunctive therapy | Time to parasitological recovery: Time (in hours) to clearance of parasitemia from the blood (both 50% and 90% decrease from admission baseline value). Parasitemia will be quantified at admission and every 6h, for 4 days or until 2 negative readings are reported. |
| Clearance of the parasite [ Time Frame: 3 days ]^17^ | Primary | Anti-malarial therapy | Clearance of the parasite [Time Frame: 3 days] |
| Parasite clearance time [ Time Frame: 7 days ]^19^ | Secondary | Adjunctive therapy | Parasite clearance time assessed by microscopy of peripheral blood films will be assessed every 6hours for the presence of asexual parasitaemia until negative on 2 consecutive blood films. Parasite half lives and clearance time will be compared between groups. Parasites will also be staged to assess if sequestration is inhibited due to temperature reduction. |
| Time to parasite clearance [ Time Frame: From admission to approximately 72 hours]^21^ | Secondary | Adjunctive therapy | Parasitological efficacy outcome; quantitative assessment of parasite density by light microscopy of Giemsa-stained thin smears. |
| Parasite clearance time [ Time Frame: 24 hours ]^24^ | Primary | Anti-malarial therapy | The proportion of patients with parasite clearance (>99% reduction from Baseline) at 24hrs after initiation of study drug. |
| Parasite clearance time [ Time Frame: 24 hours ]^27^ | Secondary | Adjunctive therapy | Parasite clearance time was defined as the time from the start of a patient’s treatment until the patient’s first negative blood film, with the blood film then remaining negative for 24 hours. |

**References:**

1. Abdallah TM, Elmardi KA, Elhassan AH, Omer MB, Elhag MS, Desogi MA. Comparison of artesunate and quinine in the treatment of severe Plasmodium falciparum malaria at Kassala hospital, Sudan. J Infect Dev Ctries. 2014;8(5):611–5.

2. Akech SO, Jemutai J, Timbwa M, Kivaya E, Boga M, Fegan G, et al. Phase II trial on the use of Dextran 70 or starch for supportive therapy in Kenyan children with severe malaria. Crit Care Med. 2010;38(8):1630–6.

3. Byakika-Kibwika P, Achan J, Lamorde M, Karera-Gonahasa C, Kiragga AN, Mayanja-Kizza H, et al. Intravenous artesunate plus Artemisnin based Combination Therapy (ACT) or intravenous quinine plus ACT for treatment of severe malaria in Ugandan children: A randomized controlled clinical trial. BMC Infect Dis. 2017;17(1).

4. Dondorp AM, Fanello CI, Hendriksen IC, Gomes E, Seni A, Chhaganlal KD, Bojang K, Olaosebikan R, Anunobi N, Maitland K, Kivaya E, Agbenyega T, Nguah SB, Evans J, Gesase S, Kahabuka C, Mtove G, Nadjm B, Deen J, Mwanga-Amumpaire J, Nansumba M, Karema C U. Artesunate versus quinine in the treatment of severe falciparum malaria in African children (AQUAMAT): an open-label, randomised trial. Lancet. 2010; 376(9753):1647–57.

5. Eltahir HG, Omer AA, Mohamed AA, Adam I. Comparison of artesunate and quinine in the treatment of Sudanese children with severe Plasmodium falciparum malaria. Trans R Soc Trop Med Hyg. 2010;104(10):684–6.

6. Maude RJ, Hoque G, Hasan MU, Sayeed A, Akter S, Samad R, Alam B, Yunus EB, Rahman R, Rahman W, Chowdhury R, Seal T, Charunwatthana P, Chang CC, White NJ, Faiz MA, Day NP, Dondorp AM HA. Timing of enteral feeding in cerebral malaria in resource-poor settings: a randomized trial. PLoS One. 2011;6(11:e27273).

7. Maude RJ, Silamut K, Plewes K, Charunwatthana P, Ho M, Abul Faiz M, et al. Randomized controlled trial of levamisole hydrochloride as adjunctive therapy in severe falciparum malaria with high parasitemia. J Infect Dis. 2014;209(1):120–9.

8. Mwanga-Amumpaire J, Ndeezi G, Tumwine JK. Original articles effect of vitamin A adjunct therapy for cerebral malaria in children admitted to Mulago hospital: A randomized controlled trial. Afr Health Sci. 2012;12(2):90–7.

9. Yeo TW, Lampah DA, Rooslamiati I, Gitawati R, Tjitra E, Kenangalem E, et al. A Randomized Pilot Study of L-Arginine Infusion in Severe Falciparum Malaria: Preliminary Safety, Efficacy and Pharmacokinetics. PLoS One. 2013;8(7).

10. ClinicalTrials.gov [Internet]. Effect of Paracetamol on Kidney Function in Severe Malaria (PROTECtS). 2020; Available from: https://clinicaltrials.gov/ct2/show/NCT04251351. Identifier: NCT04251351. Accessed 10 July 2020. [Internet]. [cited 2020 Jul 10]. Available from: https://clinicaltrials.gov/ct2/show/NCT04251351

11. ClinicalTrials.gov [Internet]. Treating Brain Swelling in Pediatric Cerebral Malaria. 2018 ; Available from: https://clinicaltrials.gov/ct2/show/NCT03300648. Identifier: NCT03300648. Accessed 10 July 2020.

12. ClinicalTrials.gov [Internet]. Effect of Paracetamol on Renal Function in Plasmodium Knowlesi Malaria (PACKNOW). 2016; Available from: https://clinicaltrials.gov/ct2/show/NCT03056391. Identifier: NCT03056391. Accessed 10 July 2020.

13. ClinicalTrials.gov [Internet]. Rosiglitazone Adjunctive Therapy for Severe Malaria in Children. 2016; Available from: https://clinicaltrials.gov/ct2/show/NCT02694874. Identifier: NCT02694874. Accessed 10 July 2020. In.

14. ClinicalTrials.gov [Internet]. A Trial of the Efficacy of Artesunate and Three Quinine Regimens in the Treatment of Severe Malaria in Children at the Ebolowa Regional Hospital - Cameroon. 2013; Available from https//clinicaltrials.gov/ct2/show/NCT02563704 Identifier NCT02563704 Accessed 10 July 2020.

15. ClinicalTrials.gov [Internet]. Bio-avacilability of Rectal Artesunate in Children with Severe Falciparum Malaria (REACH). Available from: https://clinicaltrials.gov/ct2/show/NCT02492178. 2015; Identifier: NCT02492178. Accessed 10 July 2020.

16. ClinicalTrials.gov [Internet]. A Safety and Feasibility Study of Enteral LVT vs. Standard of Care for Seizure Control in Pediatric CM (LVT2). 2014; Available from: https://clinicaltrials.gov/ct2/show/NCT01982812. Identifier: NCT01982812. Accessed 10 July . (Standard of Care for Seizure Control in Pediatric CM).

17. ClinicalTrials.gov [Internet]. Intravenous Artesunate and Malaria (IVAS). 2013; Available from: https://clinicaltrials.gov/ct2/show/NCT01805232. Identifier: NCT01805232. Accessed 10 July 2020.

18. ClinicalTrials.gov [Internet]. Acute vs. Delayed Iron: Effect on Red Cell Iron Incorporation in Severe Malaria. 2013; Available from: https://clinicaltrials.gov/ct2/show/NCT01754701. Identifier: NCT01754701. Accessed 10 July 2020.

19. ClinicalTrials.gov [Internet]. Paracetamol Effect on Oxidative Stress and Renal Function in Severe Malaria. 2012; Available from: https://clinicaltrials.gov/ct2/show/NCT01641289. Identifier: NCT01641289. Accessed 10 July 2020. In.

20. ClinicalTrials.gov [Internet]. Evaluation of the Efficacy and Safety of Inhaled Nitric Oxide as Adjunctive Treatment for Cerebral Malaria in Children. 2011; Available from: https://clinicaltrials.gov/ct2/show/NCT01388842. Identifier: NCT01388842. Accessed.

21. ClinicalTrials.gov [Internet]. Inhaled Nitric Oxide for the Adjunctive Therapy of Severe Malaria: a Randomized Controlled Trial. 2011; Available from: https://clinicaltrials.gov/ct2/show/NCT01255215. Identifier: NCT01255215. Accessed 10 July 2020. In.

22. ISRCTN registry [Internet]. The pharmacology of azithromycin in severe malaria bacterial co-infection in African children. 2017; Available from: http://www.isrctn.com/ISRCTN49726849. Identifier: ISRCTN49726849. Accessed 17 July 2020. In.

23. Pan African Clinical Trial Registry (PACTR) [Internet]. Aggressive Antipyretics for Fever Reduction in CNS Malaria. 2018; Available from: https://pactr.samrc.ac.za/TrialDisplay.aspx?TrialID=3255. Identifier: PACTR201804003255157. Accessed 30 July 2020. In.

24. Pan African Clinical Trial Registry (PACTR) [Internet]. SMAC Artesunate Follow-Up Study. 2011; Available from: https://pactr.samrc.ac.za/TrialDisplay.aspx?TrialID=0277. Identifier: PACTR201102000277177. Accessed 30 July 2020.

25. Mohanty S, Mishra SK, Patnaik R, Dutt AK, Pradhan S, Das B, et al. Brain swelling and mannitol therapy in adult cerebral malaria: A randomized trial. Vol. 53, Clinical Infectious Diseases. 2011. p. 349–55.

26. Phu NH, Tuan PQ, Day N, Mai NTH, Chau TTH, Chuong L V, et al. Randomized controlled trial of artesunate or artemether in Vietnamese adults with severe falciparum malaria. Malar J. 2010;9(97):97.

27. Treeprasertsuk S, Silachamroon U, Krudsood S, Huntrup A, Suwannakudt P, Vannaphan S, et al. Ursodeoxycholic acid and artesunate in the treatment of severe falciparum malaria patients with jaundice. J Gastroenterol Hepatol. 2010;25(2):362–8.
